# Thoroughness in Dental Operations

**Published:** 1866-04

**Authors:** W. S. Elliott

**Affiliations:** Sag Harbor, N. Y.


### Selections.

### THOROUGHNESS IN DENTAL OPERATIONS.

By W. S. ELLIOTT, D. D. S., Sag Harbor, N. Y.

The rapid advancement in dental science is made evident to the observer by the differences between the standards of excellence on the part of one and another of those operators who pronounce each and every effort good. One may perform an operation which he himself would deem *only* good, and which another would pronounce excellent, yet the superlative praise bestowed would not enhance the quality of the performance, but would rather mark the standard set up for all cases which. though pronounced excellent, would only reach mediocrity; whereas the consciousness that each operation is *only* good, tends to that increased energy which at last ends in the accomplishment of really excellent results. The disparity is made more evident as the test of time is applied, for the one who satisfies himself in pronouncing his performances good, may, sooner or later, be called upon to rectify that which was evidently a shortcoming, either through ignorance or want of skill; and on the other hand, the *only* good is followed by that which is truly most excellent. The motive in the one case as in the other is doubtless an honest one — to do all that would seem to be required for the good of the patient; yet it is not always satisfactory to those who repose confidence in that professed skill which so often controverts the results.

The young student inserts a filling which his preceptor may pronounce excellent; and such it may be under the circumstance of his limited knowledge and experience. The measure of praise is not unduly extended, for comparison is made to a standard temporarily set which to the beginner will be at least appreciable. As knowledge is acquired and experience gained, the standard is raised, and that effort which was looked upon as especially meritorious, is now regarded as perhaps quite defective in many essential particulars. Where the motive then is known to be unquestioned, the want of complete success is referable only to the want of knowledge of what is required in any given case; and he who assumes the practice of dentistry without that tuition which is capable of working up to the highest standard of excellence, will fall far short of what is required of him, and at the same time necessarily to the disparagement of his own interests.

How then shall the desired end be gained? The facilities for professional education in this country are so ample, that excuses for foregoing the advantages presented are utterly invalid, and the student who has served his term of private study, need not be thwarted in his purpose of honorably practicing his chosen and most worthy calling.

Empiricism and mountebankery rear their hideous forms from the undergrowth of ignorance all over this wide land, and would seem to prosper—to those who are struggling from day to day, honestly, earnestly, and intelligently to obtain a livelihood and achieve a worthy reputation. But the mountebank show is false, it is glossed over with the gold from the pockets of many a worthy and confiding patient, who will, most assuredly, pronounce the dictum upon that avaricousness and dishonesty which clutches at their hard-earned gains with pretentious assumptions. Then, to those who would eschew this class of operators and would rise above the level of mediocrity, I would say, read, study, write; and when opportunity presents, fall into the line of noble pioneers who are working, laboring, striving for your good.

The basal knowledge as offered by our collegiate institutions being attained, then work—work with a will to gain that goal planted by your Alma Mater. Be honest, be true.

But it was not my design to offer a dissertation upon the moral obligations of the dental practioner, but to make reference to a few of the causes of failure which are daily brought to my observation.

The operation of filling a cavity, I deem, is not necessarily complete, until the visual causes which would promote further decay are removed; for instance, a spongy or fungus gum approximates a cavity of decay to that extent that it is necessary to wedge it back in order to gain access, and, though the operation of filling is skillfully performed, a degree of failure may be anticipated by allowing the gum to return over a portion of the filling, thereby forming a receptacle for the retention of depraved mucus and other foreign matter. This is but little better than a porous or spongy filling. The proper treatment of the gums is necessary to complete success.

Again, in reference to the treatment and filling of pulp cavities which have remained open for an indefinite period.

To fill such teeth upon first presentation, is almost certainly to excite the periosteum to a degree of congestion or inflammation ; and too great haste in the treatment thereof will tend to similar results. In most cases of failure it will be proven that either sufficient time or care was not taken in the preparation of the cavity ; that the dentinal exudates were turned back too suddenly upon the investing membrane, thereby causing irritation. My usual plan is to thoroughly cleanse the cavity by excavating and syringing with cold water at the first sitting. In about six hours, syringe again and saturate with creosote, leaving the cavity open. The next day syringe —always with cold water—and apply cotton and creosote, which, after three or four hours, may be removed, the cavity left open a few minutes, then another pellet of cotton slightly moistened with creosote may be packed carefully but firmly into the root and covered with gutta-percha. In one week, if all has gone right, a permanent crown-filling may be inserted over the gutta percha. This plan, in my own practise, is generally attended with success.

Another common cause of failure, in the filling of approximal cavities, is a want of thoroughness in excavating that portion of the dentine nearest the neck of the tooth. This is particularly so when there is a spongy condition of the gums, which, as before observed, should be first restored to perfect health.

Many fillings too are failures from the imperfect condensation of the first portions of gold introduced. After anchoring the initial pellet, the utmost care should be taken that it does not become moved from its position. To prevent which, it is desirable to have an assistant who will press this portion of the gold to its place, while the operater proceeds to weld to the piece thus secured.

Finally, let each successive step in the operation be thorough, and you will have the satisfaction of knowing that the result is really excellent.

